# Short inter-pregnancy interval and birthweight: a reappraisal based on a follow-up study of all women in Norway with two singleton deliveries during 1970–2019

**DOI:** 10.1007/s10654-024-01148-y

**Published:** 2024-08-24

**Authors:** Anne Eskild, Irene Skau, Camilla Haavaldsen, Ola Didrik Saugstad, Jostein Grytten

**Affiliations:** 1 Division of Obstetrics and Gynaecology, Akershus University Hospital, Lørenskog, 1478 Norway; 2 Institute of Clinical Medicine, University of Oslo, Oslo, Norway; 3 Department of Community Dentistry, University of Oslo, Oslo, Norway; 4 Department of Paediatric Research, Oslo University Hospital, Oslo, Norway

**Keywords:** Pregnancy, Birthweight, Risk factors, Inter-pregnancy interval, Cohort study, Gestational age, Recurrence risk

## Abstract

We studied mean changes in birthweight from the first to the second delivery according to length of the inter-pregnancy interval. We also studied recurrence risk of low birthweight, preterm birth and perinatal death. We followed all women in Norway from their first to their second singleton delivery at gestational week 22 or beyond during the years 1970–2019, a total of 654 100 women. Data were obtained from the Medical Birth Registry of Norway. Mean birthweight increased from the first to the second delivery, and the increase was highest in pregnancies conceived < 6 months after the first delivery; adjusted mean birthweight increase 227 g (g) (95% CI; 219–236 g), 90 g higher than in pregnancies conceived 6–11 months after the first delivery (137 g (95% CI; 130–144 g)). After exclusion of women with a first stillbirth, the mean increase in birthweight at inter-pregnancy interval < 6 months was attenuated (152 g, 95% CI; 143–160 g), but remained higher than at longer inter-pregnancy intervals. This finding was particularly prominent in women > 35 years (218 g, 95% CI; 139 –298 g). In women with a first live born infant weighing < 2500 g, mean birthweight increased by around 1000 g to the second delivery, and the increase was most prominent at < 6 months inter-pregnancy interval. We found increased recurrence risk of preterm birth at inter-pregnancy interval < 6 months, but no increased recurrence risk of low birthweight, small for gestational age infant or perinatal death. In conclusion, we found the highest mean increase in birthweight when the inter-pregnancy interval was short. Our results do not generally discourage short pregnancy intervals.

**Supplementary Information:**

The online version contains supplementary material available at 10.1007/s10654-024-01148-y.

## Introduction

A short inter-pregnancy interval is reported to increase the risk of giving birth to an infant with low birthweight and also increase the risk of several other adverse pregnancy outcomes [1–7]. Low birthweight has been associated with infant death and impaired health in childhood and adult life [8, 9]. Also within normal ranges, birthweight has been inversely linked to risk of neonatal death and to all-cause mortality in adult life [9–11]. It is not known whether birthweight within normal ranges is associated with the length of the inter-pregnancy interval.

Recent studies suggest that the association of short inter-pregnancy interval with adverse pregnancy outcomes is attenuated or absent if differences between successive pregnancies within the same women are studied as opposed to differences between all first and second deliveries [12, 13]. Insufficient control for time dependent confounders may also have biased previous estimates [14]. Thus, a causal relation of short inter-pregnancy interval with low birthweight and other adverse pregnancy outcomes has been questioned [15, 16].

The World Health Organization (WHO) recommends women to space their childbirths, and at least 24 months inter-pregnancy interval is suggested to optimize the health of the child [17]. In many developed countries mean age of first-time mothers now exceeds 30 years, and a large proportion is 35 years or older [18]. Waiting two-three years before attempting a new pregnancy may seem long for a 35-year-old first time mother who wants more than one child. It is well known that the chances of a successful pregnancy decreases by the woman’s age [19]. Women want to optimize their children’s health, but they also want to optimize their chances of a successful new pregnancy. Thus, women need reliable information about the risks in a new pregnancy if the inter-pregnancy is short. A recent study suggests that the risk of adverse pregnancy outcomes at short intervals is lowest among women with highest reproductive age [7].

Interestingly, the risk of preeclampsia and recurrent stillbirth seem to be lowest if the inter-pregnancy interval is short [20, 21]. These pregnancy complications are linked to low infant birthweight [22]. Recent studies suggest that the chance of a successful pregnancy after a miscarriage is highest if the inter-pregnancy interval is short [23–25]. These findings suggest that a short inter-pregnancy interval may be advantageous for some outcomes.

Our main aim was to study the relation of inter-pregnancy interval with changes in birthweight from the first to the second delivery. We followed all women in Norway from their first singleton delivery at gestational week 22 or beyond during the years 1970–2009, until a consecutive singleton delivery before 2020. Among women with a first live born child, we made separate analyses according to their age at the first delivery. We also studied mean increase in birthweight after a first infant with birthweight less than 2500 g, mean increase in gestational age at birth, recurrence risk of low birthweight, preterm birth, and perinatal death according to inter-pregnancy interval.

## Materials and methods

### Cohort selection

We performed a prospective study, and we included all women in Norway with a first and a second consecutive delivery of a singleton infant (stillborn or live born) at gestational week 22 or beyond. Women with a first delivery during the years 1970–2009 were included, and they were followed until 2020 allowing all women in the study to have an inter-pregnancy-interval of at least 10 years (120 months). The women were followed from the first to the second delivery by using data from the Medical Birth Registry of Norway. The Medical Birth Registry of Norway includes information about all births in Norway after 16 weeks of gestation since 1967. Reporting of births is compulsory for the midwife or the doctor who attend the delivery [26]. The women were followed from the first to the second delivery by using their unique person identification number in the Medical Birth Registry.

In total, 731 612 women had at least two singleton deliveries during our study period (Supplemental Fig. 1). We excluded women with one or two pregnancies after in vitro fertilization (IVF) (*n* = 8066) since birthweight and inter-pregnancy interval in these pregnancies may differ from naturally conceived pregnancies [27]. We also excluded women without information about gestational age at delivery (*n* = 66 226) or with a pregnancy lasting less than 22 weeks (*n* = 1884). Finally, we excluded women without information about offspring birthweight (*n* = 1336). Thus, we could include 654 100 women.

### Data source

Information about all study factors was obtained from the Medical Birth Registry of Norway.

### Outcomes

Our primary outcome measure was mean change from the first to the second delivery in birthweight of the infant (in grams (g)). Firstly, we calculated the change in birthweight within each woman; as birthweight at the second delivery minus birthweight at the first delivery. To estimate mean change in the study sample as a whole, the sum of individual birthweight changes (in grams) was divided by the number of women in the sample. We also used low infant birthweight as outcome; <2500 g and *≤* 5th percentile of birthweight. The birthweight percentiles were based on the distribution of birthweight among all singleton deliveries in Norway during our study period.

Mean change in gestational age from the first to the second delivery was calculated in the same manner as change in birthweight. Additional outcomes were recurrence risk of preterm birth (delivery before gestational week 37) and recurrence risk of perinatal death (stillbirth at 22 gestational week or beyond or death during the first week after birth).

### Exposure

Inter-pregnancy interval is defined as the time from the first delivery until the onset of the second pregnancy. The onset of the second pregnancy was estimated by subtracting the duration of the second pregnancy (in number of days) from the date of the delivery. The length of the inter-pregnancy interval was grouped into six-month-intervals, 0–5, 6–11 months…, and > 124 months.

### Covariates

As potentially confounding factors, we included in the analyses maternal age at the first delivery, grouped in five-year-intervals (< 20, 20–24…, and ≥ 35 years old) [28]. We also included presence (yes/no) in the first pregnancy or the second pregnancy of any diabetes (type-1, type-2, or gestational diabetes) or any hypertension (preeclampsia, gestational hypertension, or chronic hypertension) [20, 29]. A new father to second pregnancy [30] and the mother’s country of birth (Norway, yes/no) [31] were also included as potentially confounding factors. To adjust for possible changes in the composition of pregnancies or in health care during our study period, we included in the data analyses year of the first delivery (in five periods with equal number of women (quintiles)) [32].

Gestational age at delivery (in days) was estimated from the date of last menstrual period for births during the years 1970–1998. After 1999, gestational age at delivery was based on foetal size at routine foetal ultrasound examination 17–19 weeks after the last menstrual period (for 98% of these pregnancies).

The Medical Birth Registry holds information about smoking since the end of 1999, and smoking may affect birthweight and inter-pregnancy interval [33]. Thus, among women with a first delivery 2000–2009 (123 170 women), we made additional adjustment for daily smoking in the first trimester or in the second pregnancy (yes/no).

### Statistical analyses

Firstly, we calculated the distribution of study factors (means and proportions) in the first and the second pregnancy. Thereafter, we estimated crude and adjusted mean changes in birthweight (in grams with 95% confidence intervals (CI)) from the first to the second delivery according to the length of the inter-pregnancy interval. We applied linear regression analyses. For crude birthweight changes, fixed effect for year of first delivery is presented. For the adjusted estimates, all the potentially confounding factors listed above were included.

We studied mean changes in birthweight in the study sample as a whole, and we repeated the analyses among women with and women without a first stillborn child. In women with a first live born child, we studied separately women aged < 25 years, 25–29 years, < 30 years, 30–35 years, and > 35 years at their first delivery, and also women with infant birthweight < 2500 g at the first delivery.

Additionally, we estimated crude and adjusted odds ratios (with 95% confidence intervals (CI)) of recurrence of birthweight; <2500 g and of small for gestational age infant (*≤* 5th percentile) according to inter-pregnancy interval.

Finally, we studied crude and adjusted mean changes in gestational age at delivery (in days) from the first to the second delivery, recurrence risk of preterm birth (< 37 gestational week), and recurrence risk of perinatal death according to the length of the inter-pregnancy interval. Recurrence risks were estimated as adjusted odd ratios (OR) with 95% CI by applying logistic regression analyses. In these analyses, inter-pregnancy interval 8–23 months is used at the reference category, and only women with the adverse outcome of interest at first delivery were included.

We used STATA version 17.0 for the statistical analyses.

## Results

Among the total of 654 100 women, 649 764 (99.3%) delivered first live born infant. Among the women with a first live born infant, mean infant birthweight was 3463 g (SD 545 g), and at the second delivery mean birthweight was 3604 g (SD 558 g) (Table 1). Mean inter-pregnancy-interval was 34.6 months (SD 29.5 months).

**Table 1:** Characteristics of the study sample at first and second delivery. All women in Norway with their first and second singleton delivery during the period 1970–2019 and first delivery < 2010 (*n* = 649 764). First stillborn exluded

Variable		First delivery		Second delivery		Change in level	
**Mean (SD)**							
	Birthweight (grams)	3463.2 (544.7)		3603.8 (557.9)		140.6	
	Gestational age at delivery (days)	281.3 (14.4)		280.7 (13.5)		-0.6	
	Inter pregnancy interval (months)					34.6 (29.5)	
**Proportion (%)**							
	Mothers age						
	< 30 years	86.5		61.7		-24.8	
	30–35 years	12.3		31.6		19.3	
	>35 years	1.2		6.7		5.5	
	Sex of child:						
	Boy	51.5		51.4		-0.1	
	Girl	48.5		48.6		0.1	
	Diabetes (any)	0.6		1.1		0.5	
	Preeclampsia/hypertension	6.2		3.6		-2.6	
	Daily smoking first trimester (from 1999)	16.1		10.3		-5.8	
	Same father to both pregnancies	90.1					
	Mother not born in Norway	18.1					

### Inter-pregnancy interval and birthweight

#### Mean birthweight

**Fig. 1:**
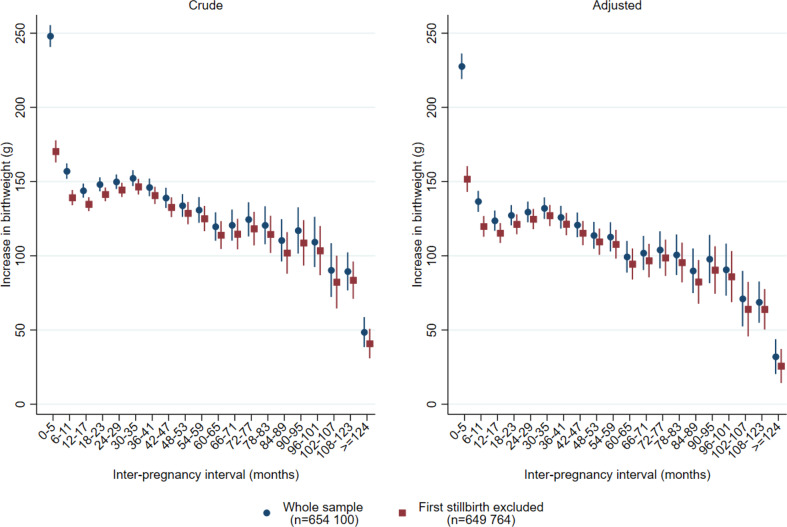
Increase in mean birthweight in grams (with 95% confidence intervals) according to the length of the inter-pregnancy interval. All women in Norway with two singleton deliveries during the period 1970-2019 and first delivery <2010. *Note* Crude estimates: Include fixed effect for year of first delivery in five periods with equal number of women (quintiles). Adjusted estimates: Include fixed effect for year of first delivery in five periods with equal number of women (quintiles), women’s age at the first delivery, diabetes in first or second pregnancy, hypertension in first or second pregnancy, sex of child in first and second pregnancy, a new father to the second pregnancy and whether the mother was born in Norway.

The increase in birthweight from the first to the second delivery was most prominent if the interval between the pregnancies was short (Fig. 1, Supplemental Table 1). In pregnancies conceived < 6 months after the first delivery, adjusted mean increase in birthweight was 227 g (95% CI; 219 –236 g), 90 g higher than in pregnancies conceived 6–11 months after the first delivery (137 g (95% CI; 130–144 g). After exclusion of women with stillbirth at the first delivery, the adjusted mean increase in birthweight at inter-pregnancy intervals < 6 months was attenuated (152 g, 95% CI; 143 –160 g), but remained higher than at longer inter-pregnancy intervals (Fig. 1, Supplemental Table 1). We found no difference in adjusted birthweight increase across inter-pregnancy intervals 6 to 59 months. However, by increasing length of the interval, the point estimated difference in birthweight between the first and the second pregnancy declined. At inter-pregnancy intervals > 124 months, the adjusted mean increase in birthweight was 25 g (95% CI; 14 –37 g). The pattern of highest birthweight increase at the shortest inter-pregnancy interval was observed in each decade during our observation period (Supplemental Fig.  2). Additional adjustment for smoking did not change the estimates notably (smoking information available since 2000) (Supplemental Fig.  3).

**Fig. 2:**
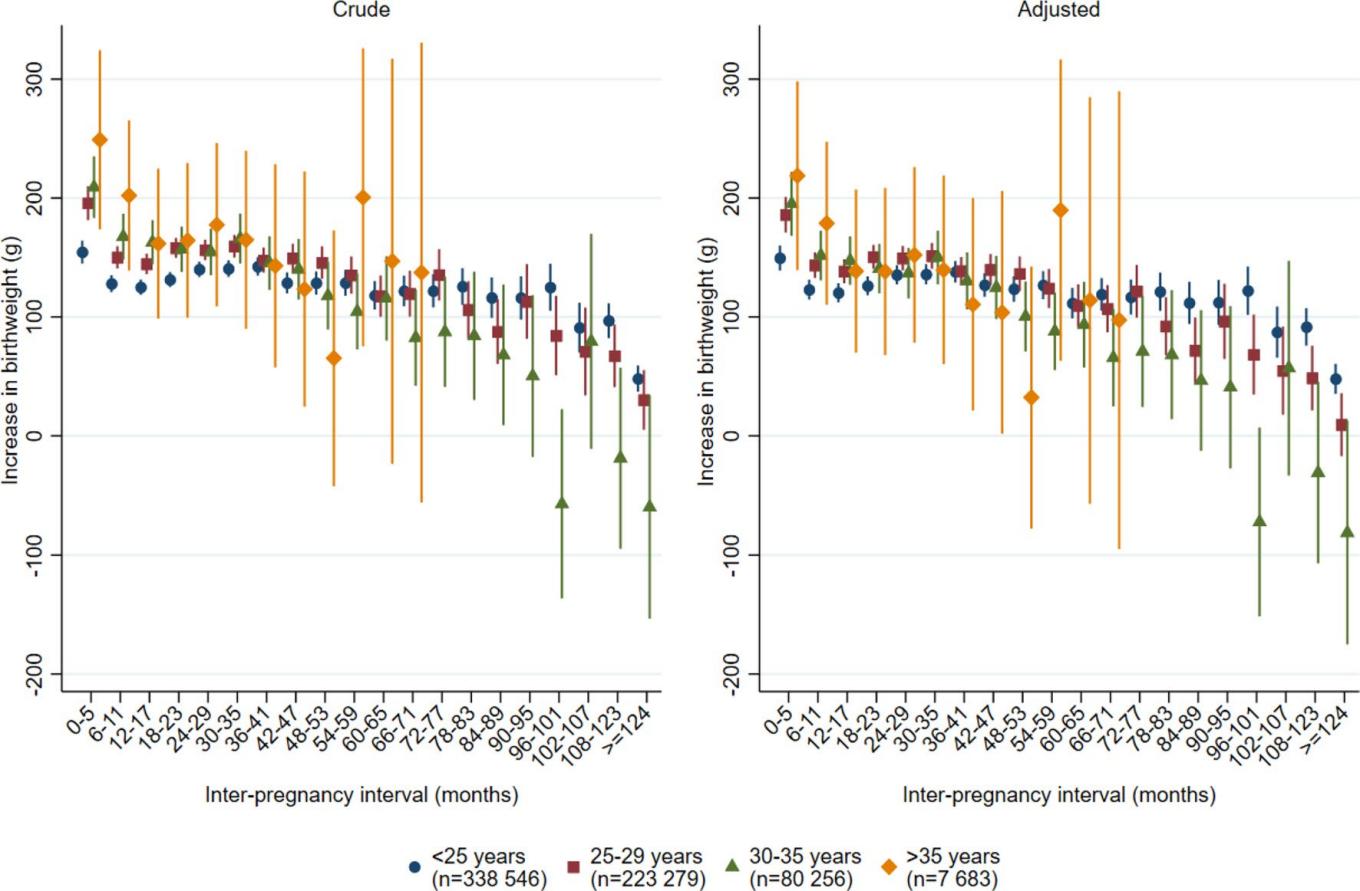
Increase in mean birthweight in grams according to inter-pregnancy interval and women’s age at first delivery. All women in Norway with two singleton deliveries during the period 1970-2019 and first delivery <2010 First stillbirth excluded. *Note* Crude estimates: Include fixed effect for year of first delivery in five periods with equal number of women (quintiles). Adjusted estimates: Include fixed effect for year of first delivery in five periods with equal number of women (quintiles), mother’s age at the first delivery, diabetes in first or second pregnancy, hypertension in first or second pregnancy, sex of child in first and second pregnancy, a new father to the second pregnancy and whether the mother was born in Norway. Intervals up to 72 months for mothers aged >35 years

Interestingly, women older than 35 years had the highest point estimated mean increase in birthweight from the first to the second delivery (218 g, 95% CI; 139 –298 g) at inter-pregnancy intervals < 6 months (Fig. 2, Supplemental Table 2). The corresponding increase among women aged 30–35 years was 195 g (95% CI; 168 –222 g), and it was 150 g (95% CI 139 –1607 g) among women less than 25 years old.

**Fig. 3:**
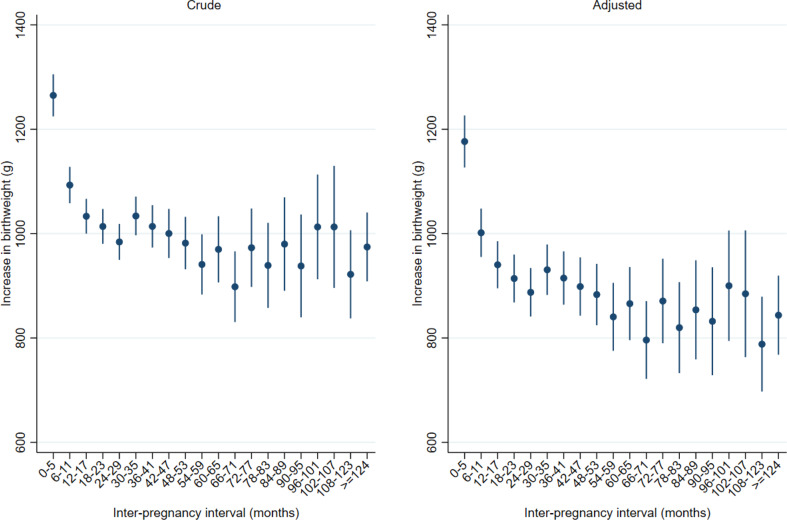
Increase in mean birthweight in grams according to inter-pregnancy interval. Birthweight at first delivery <2500 g. Two singleton deliveries during the period 1970-2019 and first delivery <2010 (n=25 369). First stillbirth excluded. *Note* Crude estimates: Include fixed effect for year of first delivery in five periods with equal number of women (quintiles). Adjusted estimates: Include fixed effect for year of first delivery in five periods with equal number of women (quintiles), women’s age at the first delivery, diabetes in first or second pregnancy, hypertension in first or second pregnancy, sex of child in first and second pregnancy, a new father to the second pregnancy and whether the mother was born in Norway

At first delivery, 25 367 women (3.6%) gave birth to a live born infant with birthweight < 2500 g. Among these women, the adjusted mean increase in birthweight was highest among women with inter-pregnancy interval < 6 months (1177 g, 95% CI; 1127 –1226 g) (Fig. 3, Supplemental Table 3). At inter-pregnancy intervals 6–11 months, mean birthweight increase was 1002 g (95% CI 955 –1048 g). At inter-pregnancy intervals beyond 11 months, the birthweight increase had overlapping confidence intervals.

### Recurrence of low birthweight

Among the 25 369 women with birthweight < 2500 g at the second delivery, 3796 women experienced recurrence of low birthweight (15%). We estimated no differences in recurrence risk for birthweight < 2500 g according to inter-pregnancy interval (Supplemental Fig.  4). The point estimated recurrence risk for a small for a gestational age infant (< 5th percentile) was similar across all inter-pregnancy intervals (Supplemental Fig.  4).

### Inter-pregnancy interval and gestational age at delivery

#### Mean gestational age

**Fig. 4:**
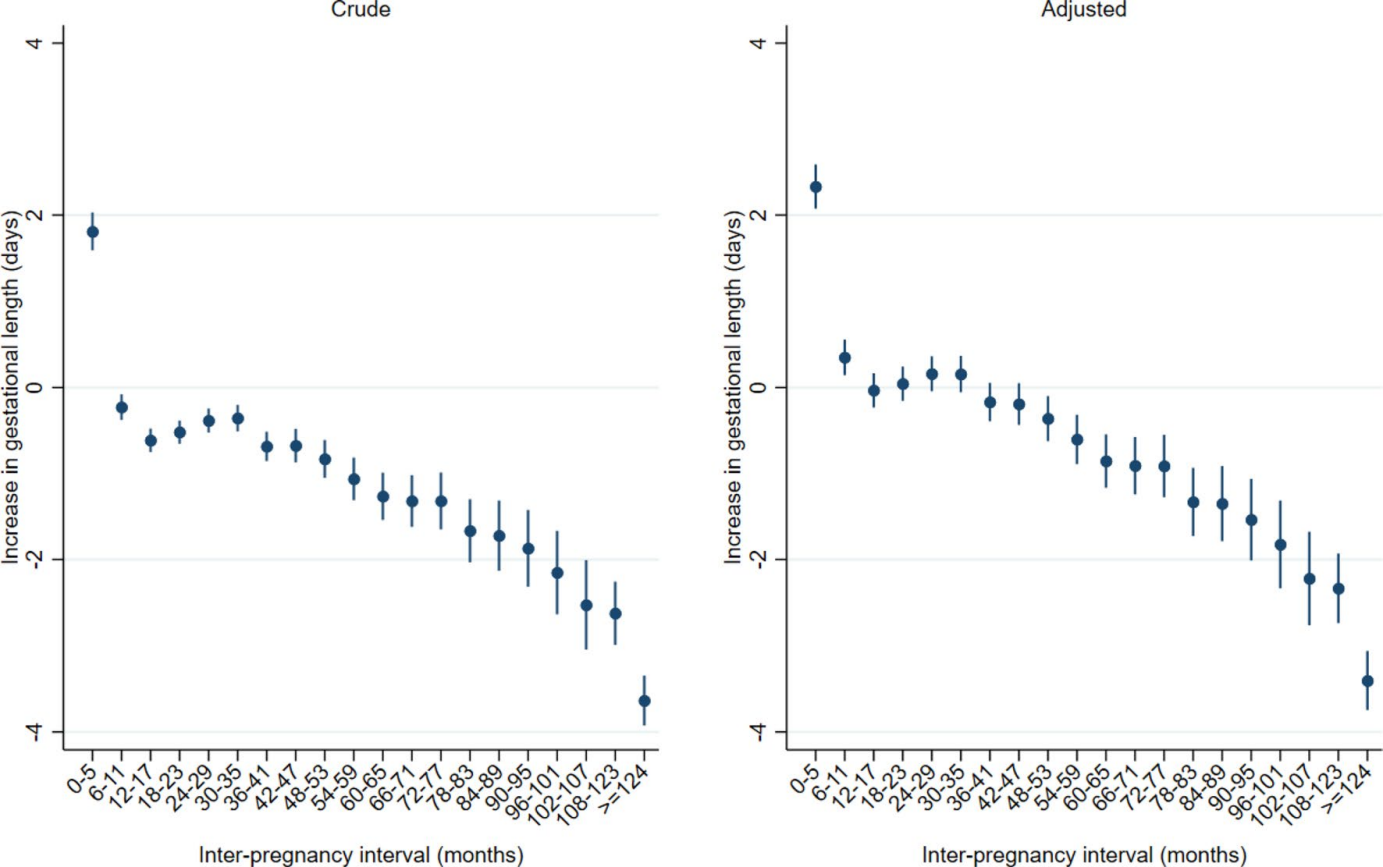
Increase in mean gestational age at delivery (in days) according to inter-pregnancy interval. All women in Norway with two singleton deliveries during the period 1970-2019 and first delivery <2010. First stillbirth excludeds. *Note* Crude estimates: Include fixed effect for year of first delivery in five periods with equal number of women (quintiles).Adjusted estimates: Include fixed effect for year of first delivery in five periods with equal number of women (quintiles), women’s age at the first delivery, diabetes in first or second pregnancy, hypertension in first or second pregnancy, sex of child in first and second pregnancy, a new father to the second pregnancy and whether the woman was born in Norway

At inter-pregnancy intervals < 6 months, the adjusted mean increase in gestational age from the first to the second delivery was 2.3 days (95% CI; 2.1 days − 2.5 days) (Fig. 4, Supplemental Table 4). There was no difference in gestational age between first and second delivery at inter-pregnancy intervals between 6 and 35 months. However, the point estimates were decreasing, and at inter-pregnancy intervals > 36 months mean gestational age was lower than at the first delivery. At inter-pregnancy intervals > 124 months, gestational age was 3.6 days lower (95% CI; -3.7 to -3.1 days) than at the first delivery.

### Recurrence of preterm birth

Among the 34 944 women with a first preterm delivery of a live born infant, 5714 women (16%) experienced recurrent preterm birth. The point estimated recurrence risk of preterm birth, displayed a j-shape curve with higher recurrence risk at < 6 months inter-pregnancy interval than at 6–11 months (Supplemental Fig.  4). Beyond 11 months inter-pregnancy interval, the point estimated recurrence risk of preterm delivery showed an increasing trend. Among the women with recurrent preterm birth and inter-pregnancy interval < 6 months, mean gestational age increased from the first to the second delivery from 225 days to 235 days, and mean birthweight increased from 1975 g to 2386 g (data not shown).

### Recurrence of perinatal death

The risk of recurrent perinatal death was low (205 out of 6376 women, 3.3%). We estimated no difference in recurrence risk according to length of the inter-pregnancy interval (Supplemental Fig.  4).

## Discussion

### Main findings

We studied mean changes in birthweight from the first to the second delivery, and we found that the increase in birthweight was highest if the inter-pregnancy interval was less than six months. This finding was more prominent among women aged 35 years or older at the first delivery than among women less than 30 years. Also among women with first live born infant weighing less than 2500 g, we found the highest increase in birthweight at inter-pregnancy intervals less than six months.

### Strengths and limitations

In our study, all first-time singleton mothers in Norway during 1970–2010 were followed at least 10 years for a second delivery to occur. Thus, we limited potential biases due to overrepresentation of women with short inter-pregnancy intervals. To our knowledge, this study is the largest yet to report the relation of inter-pregnancy interval with pregnancy outcome, and we had statistical power for separate analyses of subgroups of women. We studied changes from the first to the second delivery in a follow-up study. Such data analytic approach, reduces biases that may occur if women with an underlying risk of low birthweight or other adverse pregnancy outcomes also have a short inter-pregnancy interval [12, 13, 16].

We made adjustments for factors that have been associated with birthweight and/or the interval between pregnancies, such as maternal age at the first delivery, hypertensive disorders in any of the two pregnancies, and a new father to the second pregnancy. In a subgroup of women, we made additional adjustment for smoking. However, adjustments did not change our results notably.

Confounding may remain. Women’s body mass index may vary by the length of the inter-pregnancy interval [13, 34], and high body mass index has been associated with high offspring birthweight [35, 36]. Unfortunately, information about body mass index was available for the last two years of our inclusion period only, and half of these women lacked information. Thus, the results among these women were unreliable and therefore not presented. Both short inter-pregnancy interval and low birthweight have been associated with low socioeconomic status [37–39].It cannot be ruled out that changes in socioeconomic status from the first to the second pregnancy have confounded our results. Unfortunately, we lacked individual information about income and socioeconomic status.

Our results may not be generalizable to all women in the world [39, 40]. In addition to the high standard of living in Norway, antenatal and obstetric health care is public, free of charge and used by almost all pregnant women [41, 42]. First trimester pregnancy termination on the woman’s request has been legally performed since 1979, and pregnancy termination is free of charge. The quality and accessibility of health care may influence maternal morbidity, the interval between deliveries, birthweight, and other pregnancy outcomes. A short inter-pregnancy interval has been reported to increase maternal morbidity [43, 44].

### Interpretations of findings

It is well known that mean birthweight increases from the first to the second delivery [45, 46]. Our results suggest that the increase in birthweight from the first to the second delivery is highest if the interval is short. Birthweight is associated with future health. Birthweight, also within normal ranges, has been inversely associated with mortality [9, 10]. The higher increase in birthweight after inter-pregnancy interval < 6 months in our study may partly be explained by higher increase in gestational age at delivery (mean increase two days). Both higher birthweight and higher gestational age may be advantageous for the infant, and these outcomes are strongly interrelated.

In most previous studies, women with stillbirth were excluded. Our study illustrates that if the first infant is stillborn or has low birthweight, birthweight is likely to be higher at the second delivery particularly if the inter-pregnancy interval is short.

In most previous studies the outcome variable was dichotomous, and a short inter-pregnancy interval has been associated adverse outcomes such as low birthweight, perinatal death, preterm birth, and poor school performance of the child [2–9, 47–49]. The strength of the associations, however, has varied considerably between studies. Studies that have applied within women analyses, questions whether the increased risk of adverse outcomes at short pregnancy intervals is causal [12, 13, 21, 25].

We estimated increased recurrence risk of preterm birth at inter-pregnancy intervals < 6 months.

Among the women with recurrent preterm birth and inter-pregnancy interval < 6 months, mean increase in gestational age from the first to the second delivery was 10 days, and mean increase in birthweight was 411 g. Such increase is favourable for the infant although delivery to term could not be reached. Previously, the risk of preterm delivery according to inter-pregnancy interval has shown inconsistent results [50]. We are not aware of previous studies of recurrent perinatal death risk according to inter-pregnancy interval.

The increase in birthweight at short inter-pregnancy intervals in our study could possibly be explained by underlying factors that are linked to both short inter-pregnancy interval and high birthweight. High fecundity could be one such factor. Women with high fecundity may get pregnant shortly after a delivery, have low risk of miscarriage, and the birthweight of their offspring may be higher than women with low fecundity [34]. A biological selection to a successful pregnancy after a short inter-pregnancy interval may be particularly pronounced among women at high reproductive age [7]. An alternative hypothesis is that at long inter-pregnancy intervals, the maternal age is increased and thereby the risk is increased of maternal complications that may affect fetal growth and duration of pregnancy.

A pregnancy enhances large changes in the maternal cardiovascular system, and these changes are essential for the provision of oxygen and nutrition to the fetus [51]. The maternal adaption to pregnancy includes increased cardiac output, new vessels, increased oxygen uptake, and higher levels of growth and pro-angiogenic factors [52, 53]. It is conceivable such changes remain after a pregnancy or may easily be reactivated, particularly shortly after a previous pregnancy. A biological memory of being pregnant is supported by the low risk of preeclampsia after a short inter-pregnancy interval [20, 43], and preeclampsia is closely linked to low maternal levels of pro-angiogenic factors [54]. Increasing evidence suggests that foetal cells that are transferred to the mother during pregnancy, have multilineage differentiation capacity [55, 56], and such cells may possibly contribute to a biological memory of being pregnant.

## Conclusion

In this follow-up study of 654 100 women in Norway, we found that the increase in birthweight from the first to the second delivery was highest if the inter-pregnancy interval was less than six months. Our findings do not generally discourage a short inter-pregnancy interval.

## Electronic supplementary material

Below is the link to the electronic supplementary material.

Supplementary Material 1

Supplementary Material 2

## Data Availability

The data underlying this article may be available on request to the Medical Birth Registry of Norway.
