# Supplementary material for: Short inter-pregnancy interval and birthweight: a reappraisal based on a follow-up study of all women in Norway with two singleton deliveries during 1970–2019
Source: Eur J Epidemiol. 2024 Aug 24;39(8):905–14. doi: 10.1007/s10654-024-01148-y (PMC11410846; doi:10.1007/s10654-024-01148-y)
Supplement: Supplementary file 1 — Supplementary Material 1 [file 10654_2024_1148_MOESM1_ESM.docx]

Excluded: Women with delivery at gestational week <22 (n=1 884)

Excluding women with a first stillborn child (n=4 336)

**n= 649 764**

Women with at least two singleton deliveries 1970-2019 and first delivery <2010 at gestational week >=22, excluding pregnancies after in vitro fertilization

n=655 436

,

n=

n= 226 101

1967 - 2014

n= 1 353 979

**Final study sample:**

Women with at least two singleton deliveries 1970-2019, with first delivery <2010 at gestational week >=22 and with information about offspring birthweight excluding pregnancies after in vitro fertilization

**n= 654 100**

Final

Women with at least two singleton deliveries

1970-2019 and first delivery <2010, with

information about gestational age, excluding pregnancies after in vitro fertilization

n= 657 320

Excluded: Women with pregnancies after in vitro fertilization (n=8 066)

Excluded: Women without information about gestational age at delivery (n=66 226)

Eligible: Women with at least two consecutive singleton deliveries reported to the Medical Birth Registry of Norway, 1970-2019. First delivery <2010

n=731 612

n=

n= 226 101

1967 - 2014

n= 1 353 979

Women with at least two deliveries 1970-2019 and first delivery <2010, excluding pregnancies after in vitro fertilization

n= 723 546

n=

n= 226 101

1967 - 2014

n= 1 353 979

**Supplemental Fig. 1** Flow chart of the study sample

Excluded: Women with missing information about offspring birthweight (n=1 336)


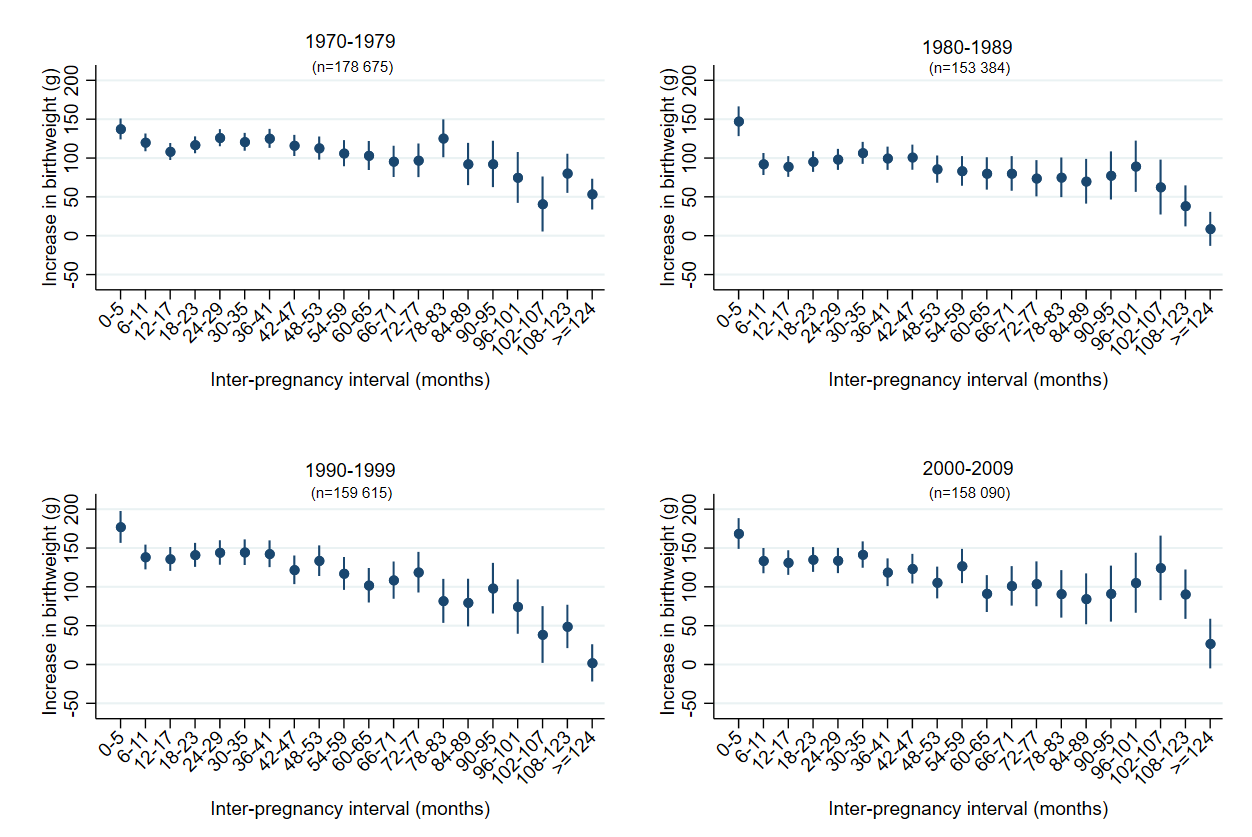


**Supplemental Fig. 2** Increase in mean birthweight in grams according to inter-pregnancy interval and decade of first delivery. First stillbirth excluded

Adjusted estimates. All women in Norway with their first and second singleton delivery during the period 1970-2019 and first delivery <2010.

NOTE: Adjusted estimates: Include fixed effect for year of first delivery in five periods with equal number of women (quintiles), women's age at the first delivery, diabetes in first or second pregnancy, hypertension in first or second pregnancy, sex of child in first and second pregnancy, a new father to the second pregnancy and whether the woman was born in Norway


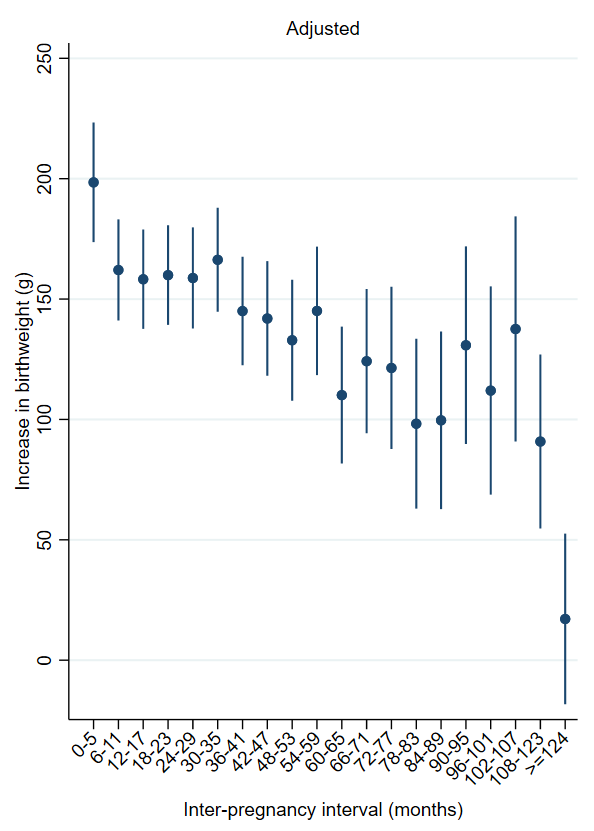


**Supplemental Fig. 3** Increase in mean birthweight in grams according to inter-pregnancy interval. First stillbirth excluded

Adjusted for daily smoking in first trimester in first or second pregnancy. Deliveries during 1999-2019 (n=123 177)

NOTE: Adjusted estimates: Include smoking, fixed effect for year of first delivery in five periods with equal number of women (quintiles), women's age at the first delivery, diabetes in first or second pregnancy, hypertension in first or second pregnancy, sex of child in first and second pregnancy, a new father to the second pregnancy and whether the woman was born in Norway

**Final study sample:**

Number of women with at least two singleton births 1970-2019, excluding pregnancies after in vitro fertilization, with pregnancies reported to last ≥ 28 weeks and with information about offspring birthweight

**n= 764 203**

Final

Excluded: Women with missing information about offspring birthweight (n=1 267)


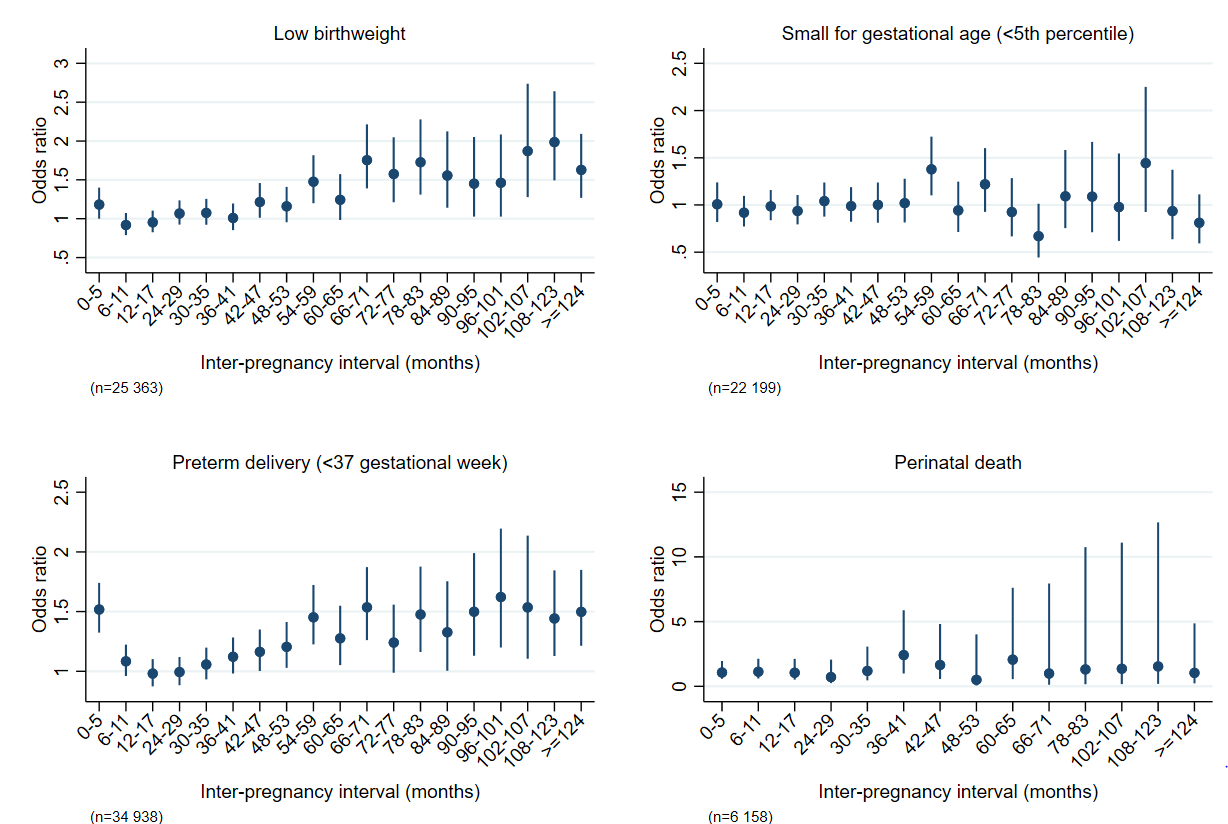


**Supplemental Fig. 4** Adjusted odds ratios for recurrence according to inter-pregnancy interval. Reference category: inter-pregnancy interval 18-23 months

First and second singleton delivery during the period 1970-2019 and first delivery <2010. First child live born, except in the analyses of recurrence of perinatal death.

NOTE: Adjusted estimates: Include fixed effect for year of first delivery in five periods with equal number of women (quintiles), women's age at the first delivery, diabetes in first or second pregnancy, hypertension in first or second pregnancy, sex of child in first and second pregnancy, a new father to the second pregnancy and whether the woman was born in Norway

**Final study sample:**

Number of women with at least two singleton births 1970-2019, excluding pregnancies after in vitro fertilization, with pregnancies reported to last ≥ 28 weeks and with information about offspring birthweight

**n= 764 203**

Final

Excluded: Women with missing information about offspring birthweight (n=1 267)
